# Supplementary material for: New Monoclonal Antibodies Specific for Different Epitopes of the Spike Protein of SARS-CoV-2 and Its Major Variants: Additional Tools for a More Specific COVID-19 Diagnosis
Source: Biomedicines. 2023 Feb 18;11(2):610. doi: 10.3390/biomedicines11020610 (PMC9953266; doi:10.3390/biomedicines11020610)

Supplementary figure S1: Schematic representation of the cloning, expression and purification of SARS-CoV-2 recombinant proteins in the HEK293T cel line

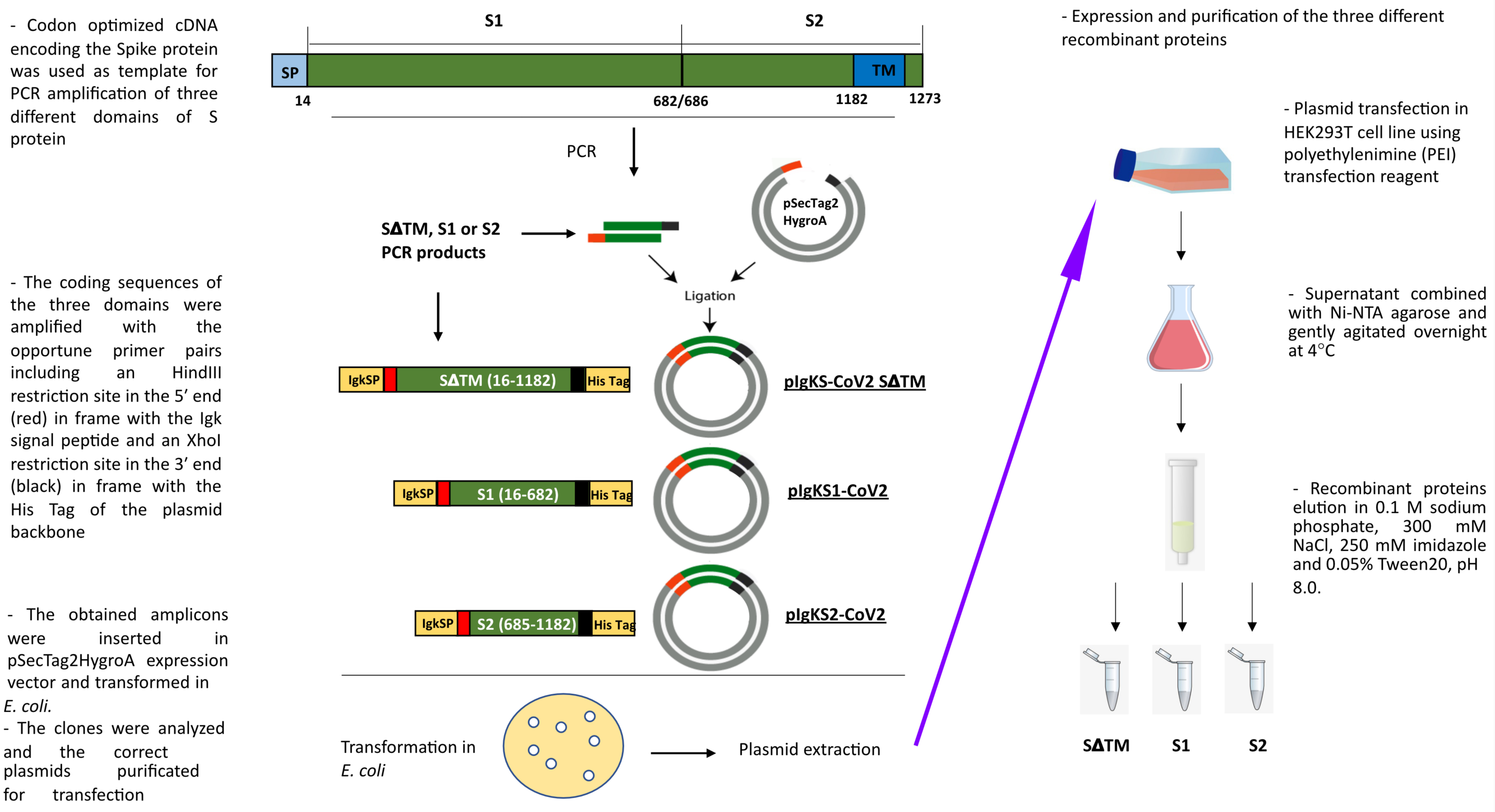

Supplement: Supplementary file 1 [file biomedicines-11-00610-s001.zip › Supplementary Fig. S1.pdf]
