# Supplementary material for: New Monoclonal Antibodies Specific for Different Epitopes of the Spike Protein of SARS-CoV-2 and Its Major Variants: Additional Tools for a More Specific COVID-19 Diagnosis
Source: Biomedicines. 2023 Feb 18;11(2):610. doi: 10.3390/biomedicines11020610 (PMC9953266; doi:10.3390/biomedicines11020610)
Supplement: Supplementary file 1 [file biomedicines-11-00610-s001.zip › Supplementary Fig. S2.pdf]

Figure S2. Clustal Omega multiple sequence alignment. Aminoacidic sequence of SARS-CoV-2 spike protein (NCBI accession number YP_009724390) was compared to that of alpha-HCov 229E (NCBI accession number NP_073551.1) and NL63 (NCBI accession number BBL54116.1), beta-HCov OC43 (NCBI accession number AOL02453.1) and HKU1 (NCBI accession number AXT92561.1), MERS (NCBI accession number YP_009047204.1) and SARS-CoV (NCBI accession number AAP33697.1). Residues marked in bold correspond to epitopes recognized by mAbs described in the work. Conserved residues between Sars-Cov-2 and one or more of the others are marked in red.

229E	--	0
NL63	--mklflillilplvscfstcnsn-------------asismlqlgvpdnsstivtgllp	45
OC43	---mflillislpta--favigdl----ncpldprlkgsfnnrdtgppsistdtv-----	46
HKU1	----mfliifilptt--lavigdf----nctns-----finhynktiprisedvv-----	40
MERS	mihsvfllmflltptesyvdvgpdsvksacievdi---qqtffdktwprp----i-----	48
**SARS-CoV-2**	----mfvflvllplv-s----------sqcvn--l---tt--**rtqlppay**----------	28
SARS-CoV	----mfifllfltlt-s----------gsdld--r---cttfddvqapny---tq-----	32

<u>aa 21-40</u>
- mAb S200

229E	--	0
NL63	vhwicanqststypangffyidvgkhrsafalhsgyyda-----nqyyiyltnkislnap	100
OC43	----------dvtnglgtyyvldrvylnttlflngyyptsgstyrnm------alkgtdl	90
HKU1	----------dvslglgtyyvlnrvylnttllftgyfpksganfrdl------alkgsty	84
MERS	----------dvskadgiiypqgrtysnitityqglf-pyqgdhgdmyvysaghatgttp	97
**SARS-CoV-2**	----------**tnsftrgvyypd**kvfrssvlhstqdlflpffsnvt---wfhaihvsgtng	75
SARS-CoV	----------htssmrgvyypdeifrsdtlyltqdlflpfysnvt---gfhtin------	73

229E	--mf	2
NL63	vtlkickfgnt-sfdflsnvstshhcivnssfteqlgvplgitisgetvrlhlynatrtf	159
OC43	lst--lwfkppflsdfingifakvk----n-----tkvfkdgv---------mysefpai	130
HKU1	lst--lwykppflsdfnngifskvk----n-----tklyvnnt---------lysefsti	124
MERS	qklfvanysqd-vkqfangfvvrig----a-----aanstgtviispstsatirkiypaf	147
**SARS-CoV-2**	tkr----fdnp-vlpfndgvyfast----e----------------------ksniirgw	104
SARS-CoV	-ht----fgnp-vipfkdgiyfaat----e----------------------ksnvvrgw	101

229E	vllvaya---------------------l-lhiagcqttnglntsysvcngcvgysenvf	40
NL63	yvpaaykltkl-svkcyfsescvfsvvna-tvtvnvttlngrivnytvcddcngytdnif	217
OC43	tigstfvntsy---------svvvqprtinstqdgvnklqg-llevsvcq----------	170
HKU1	vigsvfvntsy---------tivvqph------------ng-ileitacq----------	152
MERS	mlgssvgnfsdgkmgrffnhtlvllpd-------g-----c-gtllraf-----------	183
**SARS-CoV-2**	ifgttldsktq---------sllivnn-------a-----t-nvvikvce----------	132
SARS-CoV	vfgstmnnksq---------sviiinn-------s-----t-nvviracn----------	129

229E	avesggyipsdfafnnwflltntssvvdgvvrsfqplllnclwsvsglrfttgfvyf---	97
NL63	svqqdgripngfsfnnwflltngstlvdgvsrlyqplrltclwpvpglksstgfvyf---	274
OC43	-------------------------------------ynmcehphtichpnlgnhfk---	190
HKU1	-------------------------------------ytmceyphtvcrsk-gsirn---	171
MERS	--------------------------------------ycileprsgnhcpagnsytsfa	205
**SARS-CoV-2**	-------------------------------------fqfcndpflgvyyhknn------	149
SARS-CoV	-------------------------------------felcdnpffavskpmgt------	146

229E	--ngtgr-gdckgfssd-vlsd----virynlnfee-------nlrrg--------tilf	134
NL63	--natgsdvncngyqhh-svad----vmrynlnfssnsv---dnlksg--------vivf	316
OC43	---------------elwhldtgvv-sclykrnftydvnatyl-------------yfhf	221
HKU1	---------------eswhidssep-lclfkknftynvsadwl-------------yfhf	202
MERS	tyhtpatdcsdgnynrnaslnsfkeyfnlrnctfmytynitedeilewfgitqtaqg-vh	264
**SARS-CoV-2**	---------------kswmesefrvyssannctfeyvsqpflmdlegkqgnfknlrefvf	194
SARS-CoV	-------------------qthtmifdnafnctfeyisdafsldvseksgnfkhlrefvf	187

229E	ktsygvvvfyctnntlv-s-----------gdahipfgtvlgnfycfvnttignettsaf	182
NL63	ktlqydvlfycsnsssg-v-----------ldttipfgpssqpyycfinstintthvstf	364
OC43	yqeggtfyayftd----------tgfvtk-flfnvylgmalshyyvmplt----------	260
HKU1	yqergvfyayyad----------vgmptt-flfslylgtilshyyvmplt----------	241
MERS	lfssryvdlyggn---------------mfqfatlpvydtikyysiiphs----------	299
**SARS-CoV-2**	knidgyfkiyskhtpinlvrdlpqgfsaleplvdlpiginitrfqtllal----------	244
SARS-CoV	knkdgflyvykgyqpidvvrdlpsgfntlkpifklplginitnfrailta----------	237

229E	vgalpktvrefv-isrtghfyingyryftlgnveavnfnvttaettdfctvalasyadvl	241
NL63	vgvlppivreiv-vartgqfyingfkyfdlgfieavnfnvttasatdfwtvafatfvdvl	423
OC43	-------ci---s--rrd-----------------------igftleywvtpltprqyll	285
HKU1	-------cn---aispkt-----------------------dnetleywvtplsrrqyll	268
MERS	-------ir---siqsdrkaw------------------------aafyvyklqpltfll	325
**SARS-CoV-2**	-------hrsyltpgdsssgw--------------------tagaaayyvgylqprtfll	277
SARS-CoV	-------fs------paqdiw--------------------gtsaaayfvgylkpttfml	264

229E	vnvsqtsianiiycn-svinrlrcdqlsfdvpdgfystspiqsvelpvsivslpvyh---	297
NL63	vnvsatniqnllycd-spfeklqcehlqfglqdgfysanflddnvlpdtyvalpiyy---	479
OC43	afnqdgiifnavdcmsdfmseikcktqsiapptgvyelngytvqpiadvyrrkpdlpncn	345
HKU1	nfdehgvitnavdcsssflseiqcktqsfapntgvydlsgftvkpvatvyrripnlpdcd	328
MERS	dfsvdgyirraidcgfndlsqlhcsyesfdvesgvysvssfeakpsgsvveqaeg-vecd	384
**SARS-CoV-2**	kyn**engtitdavdcaldplsetkctlksftvekgiyqtsnfrv**qptesivrfpnitnlcp	337
SARS-CoV	kydengtitdavdcsqnplaelkcsvksfeidkgiyqtsnfrvvpsgdvvrfpnitnlcp	324

aa 281-300
- mAb S37

aa 301-320
- mAb S75
- mAb S106

229E	-----------------khtfivlyv-----------------dfkpqsgggkcfncypa	323
NL63	-----------------qhtdinft--------------------atasfggscyvckph	502
OC43	ieawlndksvpsplnwerktfsncnfnmsslmsfiqadsftcnnidaakiygmcfssi--	403
HKU1	idnwlnnvsvpsplnwerrifsncnfnlstllrlvhvdsfscnnldkskifgscfnsi--	386
MERS	fspllsg-tppqvynfkrlvftncynltkllslfsvndftcsqispaaiasncyssl--	441
**SARS-CoV-2**	fgevfnatrfasvyawnrkrisncvadysvlynsasfstfkcygvsptklndlcftnv--	395
SARS-CoV	fgevfnatkfpsvyawerkkisncvadysvlynstffstfkcygvsatklndlcfsnv--	382

229E	gvnitlanfne---tkgplcvdtshfttkyvavya-----nvgrwsasin--t-------	366
NL63	qvnislng-------ntsvcvrtshfsiryiynrvksgspgdsswhiylk--s-------	546
OC43	----tidkfaipnrrkvdlqlgnlgylqssnyridttatscqlyynlpaanvsvsr-fnp	458
HKU1	----tvdkfaipnrrrddlqlgssgflqssnykidisssscqlyyslpllnvtinn-fnp	441
MERS	----ildyfsyplsmksdlsvssagpisqfnykqsfsnptclilatvphnlttitkplky	497
**SARS-CoV-2**	----yadsfvirgdevrqiapgqtgkiadynyklpddftgcviawnsnnldskvggnyny	451
SARS-CoV	----yadsfvvkgddvrqiapgqtgviadynyklpddfmgcvlawntrnidatstgnyny	438

229E	--gncpfsfgkvnnfvkf------------gsvcfslkdipggca-----------mpiv	401
NL63	--gtcpfsfsklnnfqkf------------kticfstvavpgscn-----------fple	581
OC43	stwnkrfgfiedsvfvpqptgvftnhsvvyaqhcfkapknfcpcss------cpgknngi	512
HKU1	sswnrrygfgsfnv---------ssydvvysdhcfsvnsdfcpcadpsvvnscvkskpls	492
MERS	syinkcsr-----llsddrtev--------pqlvn--anqyspcvs---------ivpst	533
**SARS-CoV-2**	lyrlfrks-----nlkpferdi--------steiy--qagstpcng---------vegfn	487
SARS-CoV	kyrylrhg-----klrpferdi--------snvpf--spdgkpctp----------paln	473

229E	anwayskyyti---	412
NL63	atwhytsytiv---	592
OC43	gtcpagtnyltcdn-------------lctldpitfkapdtykcpqtkslvgigehcsgl	559
HKU1	aicpagtkyrhcdldtttlyvknwcrcsclpdpisty--spntcpqkkvvvgigehcpgl	550
MERS	-vwedgdyyrk------------------qlsplegg----------------gw-----	553
**SARS-CoV-2**	cyf-----------------------------plqsy----------------gf-----	497
SARS-CoV	cyw-----------------------------plndy----------------gf-----	483

229E	-------------------gslyvswsdgdgitgv--------------pqpveg-----	434

```
NL63         --------------------galyvtwsegnsitgv--------------pypvsg----- 614
OC43         avksdycgn----nsctcqpqaflgwsadsclqgdkcnifanfilhdvnngltcstd--l 613
HKU1         gineekcgtqlnhtscscspdaflgwsfdscisnnrcnifsnfifnginsgttcsnd--l 608
MERS         ----------lvasgstvamteqlqmgfgit------------vqygtdtnsvcpklefa 591
SARS-CoV-2   ----------qptngvgyqpyrvvvlsfell----------------hapatvcgp---- 527
SARS-CoV     ----------ytttgigyqpyrvvvlsfell----------------napatvcgp---- 513

229E         vssfmnvtldkctkyniydvsgvgvirvsndtflng---itytstsgnl-lgfkdvtkgt 490
NL63         irefsnlvlnnctkyniydyvgtgiirssnqslagg---ityvsnsgnl-lgfknvstgn 670
OC43         qkanteielgvcvnydlygisgqgifvevnatyynswqnllydsn-gnly-gfrdyitnr 671
HKU1         lysntevstgvcvnydlygitgqgifkevsaayynnwqnllydsn-gnii-gfkdfltnk 666
MERS         ndtkiasqlgncveyslygvsgrgvfqnctavg-vrqqrfvyday-qnlvgyysd--dgn 647
SARS-CoV-2   -kkstnlvknkcvnfnfngltgtgvltesnkkf-lpfqqfgrdia-dtt-davrdpqtle 583
SARS-CoV     -klstdliknqcvnfnfngltgtgvltpsskrf-qpfqqfgrdvs-dft-dsvrdpktse 569

229E         iysitpcnppdqlvvyqqa------------vvgamlsenftsygfsn-----vvelpkf 533
NL63         ifivtpcnqpdqvavyqqs------------iigamtavnesryglhn-----llqlpnf 713
OC43         tfmihscysgrvsaayh--anssepallfrnikcnyvfnnsltrqlq---------piny 720
HKU1         tytilpcysgrvsaafy--qnssspallyrnlkcsyvlnnis---fi---------sqpf 712
MERS         yyclracvsvpvsviyd--ketkthatlfgsvacehisstmsqysrstrsmlkrrdstyg 705
SARS-CoV-2   ilditpcsfggvsvitpgtntsnqvavlyqdvnctevpvaihadqlt--ptwrvystgsn 641
SARS-CoV     ildispcsfggvsvitpgtnassevavlyqdvnctdvstaihadqlt--pawriystgnn 627
```

aa 550-580
- **mAb S71**
- **mAb S79**
- **mAb S178**

```
229E         fyasngtynctdavl-----------tyssfgvcadgsiia-vq---prnvsydsvs--- 575
NL63         yyvsnggnncttavm-----------tysnfgicadgslip-vr---prnssdngis--- 755
OC43         sfds--ylgcvvnaynstaisvqtcdltvgsgycvdysk--nrrsrraittgyrftn-fe 775
HKU1         yfds--ylgcvlnavnltsysvsscdlrmgsgfcidyalpssrrkrrgisspyrfvt-fe 769
MERS         plqt--pvgcvlglvnss-lfvedcklplgqslcalpdtpstltprsvrsvpgemrlasi 762
SARS-CoV-2   vfqt--ragcligaehvn--nsyecdipigagicasyqtqt-nsprrar vsvasqsi---i
SARS-CoV     vfqt--qagcligaehvd--tsyecdipigagicasyhtvs-l----lrstsqksi---v 675
```

aa 661-682
- **mAb S12**
- **mAb S157**

```
229E         -------------aivtanlsipsnwttsvqveylqitstpivvdcstyvcngnvrcvel 622
NL63         -------------aiitanlsipsnwttsvqveylqitstpivvdcatyvcngnprcknl 802
OC43         pftvnsvndslepvgglyeiqipseftignmeefiqtsspkvtidcaafvcgdyacklq 835
HKU1         pfnvsfvndsvetvgglfeiqiptnftiagheefiqtsspkvtidcsafvcsnyaachdl 829
MERS         afnhpiq-v-dqlnssyfklsiptnfsfgvtqeyiqttiqkvtvdckqyvcngfqkceql 820
SARS-CoV-2   aytmslg-aensvaysnnsiaiptnftisvtteilpvsmtktsvdctmyicgdstecsnl 752
SARS-CoV     aytmslg-adssiaysnntiaiptnfsisittevmpvsmaktsvdcnmyicgdstecanl 734
```

aa 686-700
- **mAb S136**
- **mAb S222**
- **mAb S223**

```
229E         lkqytsacktiedalrnsarlesadvsemltfdkkaftlan-----vssfgd-ynlssvi 676
NL63         lkqytsacktiedalrlsahletndvssmltfdsnafslan-----vtsfgd-ynlssvl 856
OC43         lveygsfcdninailtevnelldttqlqvanslmngvtlstklkdgvnfnvddinfspvl 895
HKU1         lseygtfcdninsilnevndllditqlqvanalmqgvtlssnlntnlhsdvdnidfksll 889
MERS         lreygqfcskinqalhganlrqddsvrnlfasvkssqsspi-----ipgfggdfnltlle 875
SARS-CoV-2   llqygsfctqlnraltgiaveqdkntqevfaqvkqiyktpp-----ikdfggf-nfsqil 806
SARS-CoV     llqygsfctqlnralsgiaaeqdrntrevfaqvkqmyktpt-----lkyfggf-nfsqil 788

229E         pslptsgsrvagrsaiedilfsklvtsglgtvdadykkct--kglsiadlacaqyyngim 734
NL63         pqrnihssriagrsaledllfskvvtsglgtvdvdykscт--kglsiadlacaqyyngim 914
OC43         gclgsecskassrsaiedllfdkvklsdvgfve-aynnct--ggaeirdlicvqsykgik 952
HKU1         gclgsqcgs-ssrslledllfnkvklsdvgfve-aynnct--ggseirdllcvqsfngik 945
MERS         p-vsistgsrsarsaiedllfdkvtiadpgymq-gyddcmqqgpasardlicaqyvagyk 933
SARS-CoV-2   p-dp---skpskrsfiedllfnkvtladagfik-qygdcl--gdiaardlicaqkfnglt 859
SARS-CoV     p-dp---lkptkrsfiedllfnkvtladagfmk-qygecl--gdinardlicaqkfnglt 841

229E         vlpgvadaermamytgsliggialggltsa----vsipfslaiqarlnyvalqtdvlqen 790
NL63         vlpgvadaermamytgsliggmvlggltsa----aaipfslalqarlnyvalqtdvlqen 970
OC43         vlppllsenqisgytlaatsaslfppwtaa----agvpfylnvqyringlgvtmdvlsqn 1008
HKU1         vlppilsetqisgyttaatvaamfppwsaa----agvpfslnvqyringlgvtmdvlnkn 1001
MERS         vlpplmdvnmeaaytssllgsiagvgwtaglssfaaipfaqsifyrlngvgitqqvlsen 993
SARS-CoV-2   vlpplltdemiaqytsallagtitsgwtfgagaalqipfamqmayrfngigvtqnvlyen 919
SARS-CoV     vlpplltddmiaaytaalvsgtatagwtfgagaalqipfamqmayrfngigvtqnvlyen 901
```

229E	qkilaasfnkamtnivdaftgvndaitqtsqalqtvatalnkiqdvvnqqgnslnhltsq	850
NL63	qkilaasfnkainnivasfssvndaitqtaeaihtvtialnkiqdvvnqqgsalnhltsq	1030
OC43	qklianafnnalhaiqqgfdatn--------------salvkiqavvnanaealnnllqq	1054
HKU1	qklianafnkallsiqngftatn--------------salakiqsvvnvnaqalnsllqq	1047
MERS	qkliankfnqalgamqtgftttn--------------eafqkvqdavnnnaqalsklase	1039
**SARS-CoV-2**	qklianqfnsaigkiqdslssta--------------salgklqdvvnqnaqalntlvkq	965
SARS-CoV	qkqianqfnkaisqiqeslttts--------------talgklqdvvnqnaqalntlvkq	947

229E	lrqnfqaisssiqaiydrldtiqadqqvdrlitgrlaalnvfvshtltkytevrasrqla	910
NL63	lrhnfqaisnsiqaiydrldsiqadqqvdrlitgrlaalnafvsqvlnkytevrssrrla	1090
OC43	lsnrfgaisaslqeilsrldaleaeaqidrlingrltalnayvsqqlsdstlvkfsaaqa	1114
HKU1	lfnkfgaissslqeilsrldnleaqvqidrlingrltalnayvsqqlsditlikagasra	1107
MERS	lsntfgaisasigdiiqrldvleqdaqidrlingrlttlnafvaqqlvrsesaalsaqla	1099
**SARS-CoV-2**	lssnfgaissvlndilsrldkveaevqidrlitgrlqslqtyvtqqliraaeirasanla	1025
SARS-CoV	lssnfgaissvlndilsrldkveaevqidrlitgrlqslqtyvtqqliraaeirasanla	1007

229E	qqkvnecvksqskrygfcgngthifsivnaapeglvflhtvllptqykdveawsglcvdg	970
NL63	qqkinecvksqsnrygfcgngthifsivnsapdgllflhtvllptdyknvkawsgicvdg	1150
OC43	mekvnecvksqssrinfcgngnhiislvqnapyglyfihfnyvptkyvtakvspglciag	1174
HKU1	iekvnecvksqsprinfcgngnhilslvqnapygllfihfsykptsfktvlvspglclsg	1167
MERS	kdkvnecvkaqskrsgfcgqgthivsfvvnapnglyfmhvgyypsnhievvsayglcdaa	1159
**SARS-CoV-2**	atkmsecvlgqskrvdfcgkgyhlmsfpqsaphgvvflhvtyvpaqeknfttapaichdg	1085
SARS-CoV	atkmsecvlgqskrvdfcgkgyhlmsfpqaaphgvvflhvtyvpsqernfttapaicheg	1067

229E	tn--gyvlrqpnlaly---kegnyyritsrimfepriptmadfvqiencnvtfvnisrse	1025
NL63	iy--gyvlrqpnlvly---sdngvfrvtsramfqprlpvlsdfvqiyncnvtfvnisrve	1205
OC43	nr--giapksgyfvnv-----nntwmytgsgyyypepitennvvvmstcavnytkapyvm	1227
HKU1	dr--giapkqgyfikq-----ndswmftgssyyypepisdknvvfmnscsvnftkapfiy	1220
MERS	nptnciapvngyfiktnntrivdewsytgssfyapepitslntkyvapqvtyqn-istnl	1218
**SARS-CoV-2**	ka---hfpregvfvsn-----gthwfvtqrnfyepqiittdntfvsgncdvvigivnntv	1137
SARS-CoV	ka---yfpregvfvfn-----gtswfitqrnffspqiittdntfvsgncdvvigiinntv	1119

229E	l--qtivpeyidvnktlqelsyklpnytvpdl-vveqynqtilnltseistlenksaeln	1082
NL63	l--htvipdyvdvnktlqefaqnlpkyvkpnf-dltpfnltylnlsselkqleaktaslf	1262
OC43	l--ntsipnlpdfkeeldqwfknqtsv-aphlsld-yinvtfldl--------------q	1269
HKU1	l--nnsipnlsdfeaefslwfknhtsi-apnltfnshinatfldl--------------y	1263
MERS	ppllgnstgidfqdeldeffknvsts-ipnfgsltqinttlldl--------------t	1263
**SARS-CoV-2**	ydplq--peldsfkeeldkyfknhtsp-dvdlgdisginasvvni--------------q	1180
SARS-CoV-1	ydplq--peldsfkeeldkyfknhtsp-dvdfgdisginasvvni--------------q	1162

229E	ytvqklqtlidninstlvdlkwlnrvetyikwpwwvwlcisvvlifvvsmlllcccstgc	1142
NL63	kttvelqglidqinstyvdlkllnrfenyikwpwwvwliisvvfvvllsllvfcclstgc	1322
OC43	vemnrlqeaikvlnhsyinlkdigtyeyyvkwpwyvwlliclagvamlvllfficcctgc	1329
HKU1	yemnviqesikslnssfinlkeigtyemyvkwpwyiwllivilfiiflmilfficcctgc	1323
MERS	yemlslqqvvkalnesyidlkelgnytyynkwpwyiwlgfiaglvalalcvffilcctgc	1323
**SARS-CoV-2**	keidrlnevaknlneslidlqelgkyeqyikwpwyiwlgfiagliaivmvtimlccmtsc	1240
SARS-CoV	keidrlnevaknlneslidlqelgkyeqyikwpwyvwlgfiagliaivmvtillccmtsc	1222

229E	cgffscfassirgcce--stklpyydvekihiq--------	1173
NL63	cgccncltssmrgccdcgstklpyyefekvhvq--------	1355
OC43	gtscf-------k--kcggccddytgyqelvik-tshdd--	1358
HKU1	gsacf-------s--kchnccdeygghndfvik-ashdd--	1352
MERS	gtncm-------gklkcnrccdryeeydlephkvhvh----	1353
**SARS-CoV-2**	csclk-------gccscgscckf-deddsepvlkgvklhyt	1273
SARS-CoV	csclk-------gacscgscckf-deddsepvlkgvklhyt	1255
