# Supplementary material for: New Monoclonal Antibodies Specific for Different Epitopes of the Spike Protein of SARS-CoV-2 and Its Major Variants: Additional Tools for a More Specific COVID-19 Diagnosis
Source: Biomedicines. 2023 Feb 18;11(2):610. doi: 10.3390/biomedicines11020610 (PMC9953266; doi:10.3390/biomedicines11020610)
Supplement: Supplementary file 1 [file biomedicines-11-00610-s001.zip › Supplementary Fig. S3.pdf]

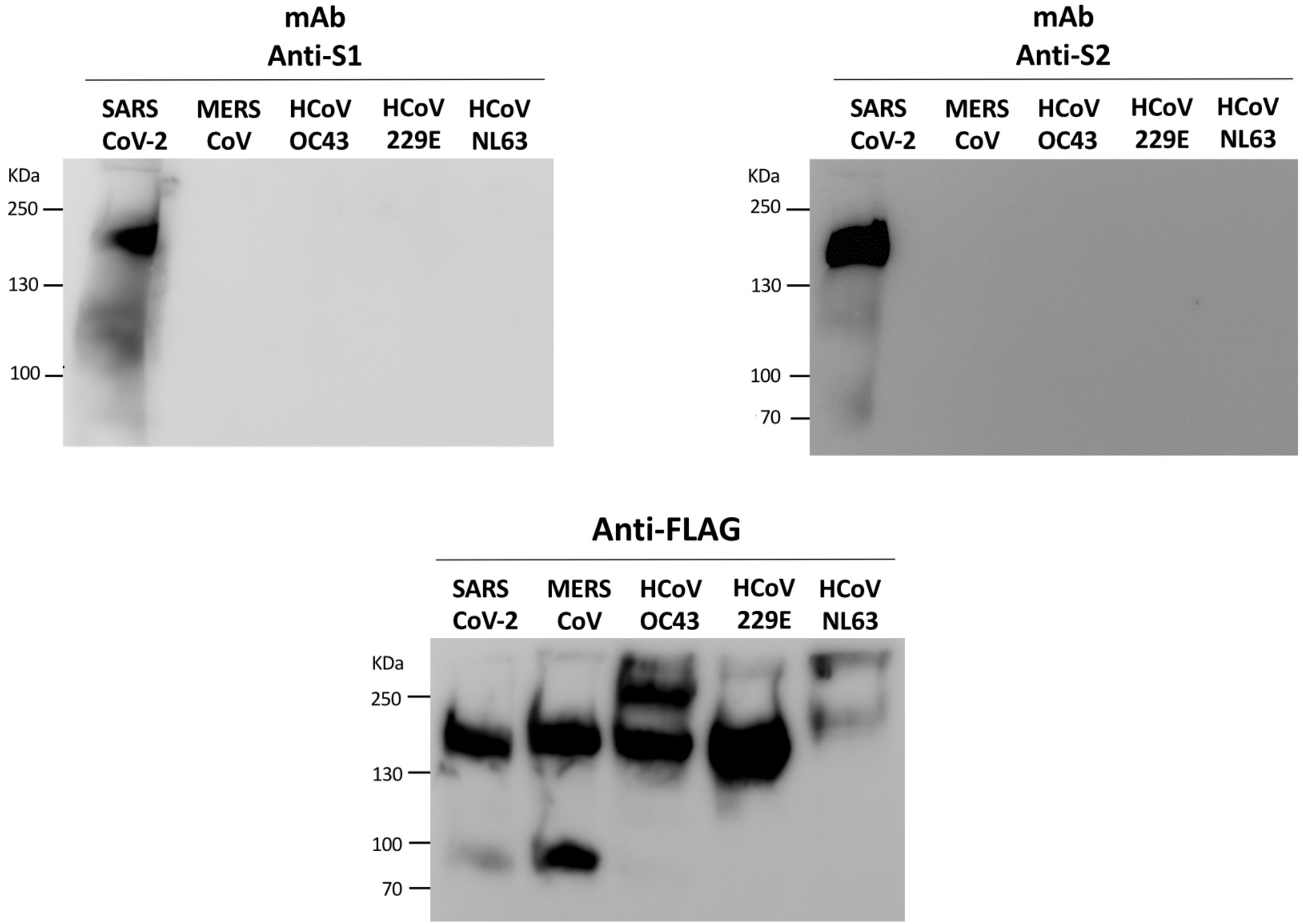

Supplementary FigS3. WB analysis of representative mAb specific for S1 domain (S79) or S2 domain (S223) and anti-FLAG as control of transfection. In SDS-PAGE cell lysates of HEK293T cells expressing the Spike protein of SARS-CoV-2, HCoV-OC43, MERS-CoV, HCoV-NL63 and HCoV-229E.
